# Serum metabolomic responses to aerobic exercise in rats under chronic unpredictable mild stress

**DOI:** 10.1038/s41598-022-09102-2

**Published:** 2022-03-22

**Authors:** Xiangyu Liu, Yumei Han, Shi Zhou, Junsheng Tian, Xuemei Qin, Cui Ji, Weidi Zhao, Anping Chen

**Affiliations:** 1 School of Physical Education, Shanxi University, Taiyuan, China; 2 Institute of Biomedicine and Health, Shanxi University, Taiyuan, China; 3 Discipline of Sport and Exercise Science, Faculty of Health, Southern Cross University, Lismore, Australia; 4 Modern Research Center for Traditional Chinese Medicine, Shanxi University, Taiyuan, China

**Keywords:** Physiology, Biomarkers, Diseases, Pathogenesis, Signs and symptoms

## Abstract

This study analyzed the effects of aerobic exercise on endogenous serum metabolites in response to chronic unpredictable mild stress (CUMS) using a rat model, aiming to identify the metabolic regulatory pathways involved in the antidepressant effect resulted from a 28-day treadmill aerobic exercise intervention. The animals were randomly divided into four groups (n = 8): normal control, normal with aerobic exercise, CUMS control, and CUMS with aerobic exercise. Body weight, sucrose preference and open field tests were performed weekly during the intervention period for changes in depressant symptoms. Serum metabolic profiles obtained by using the LC–MS/MS metabolomics were analyzed to explore the regulatory mechanism for the effect of the aerobic exercise on depression. Behavior tests showed that the aerobic exercise resulted in a significant improvement in depression-like behavior in the CUMS rats. A total of 21 differential metabolites were identified as being associated with depression in serum metabolic profile, of which the aerobic exercise significantly modulated 15, mainly related to amino acid metabolism and energy metabolism. Collectively, this is the first study that LC–MS/MS techniques were used to reveal the modulatory effects of aerobic exercise on the serum metabolic profile of depressed rats and the findings further enriched our understanding of potential mechanisms of aerobic exercise interventions on depression.

## Introduction

Depression is a common mental health disorder and can cause great suffering to the patient^1^. The World Health Organization (WHO) lists depression as a leading cause of disability worldwide and a major contributor to the overall global burden of diseases. The prevalence of depression is expected to jump to the top of the list of disorders by 2030^2^. The etiology of depression is complex and the pathogenesis remains unclear to date. Animal models using chronic unpredictable mild stress (CUMS) have been frequently utilized in research on depression^3,4^. The CUMS causes animals to develop a range of depression-like behavioral and physiological responses that are similar to depressive symptoms in humans^5^.

Currently, medication is still the primary treatment for depression, but medication can cause some adverse reactions, and has a low recovery rate after stopping medication, a high relapse rate, and a high cost^6,7^. Exercise interventions, particularly aerobic exercise, have received increasing attention as they can effectively alleviate symptoms of depression, improve physical health and fitness, and with little side effect and low cost^8^. Studies have demonstrated that aerobic exercise is effective in the prevention and treatment of depression and can significantly improve the mood of patients with major depression in a short period, especially effective for mild to moderate depression^9^. The antidepressant effect of aerobic exercise is found to be comparable to that of antidepressant drugs^10^.

Numerous studies suggest that the onset and development of depression are closely related to the metabolic disorders of endogenous metabolites^11,12^. As an important method of system biology research, metabolomics mainly analyzes small molecule metabolites with molecular weight below 1000 Da^13^, which can simultaneously monitor multiple metabolic pathways and infer the regulation process of metabolic pathways through the upregulation or downregulation of landmark metabolites to explore the pathological mechanism of diseases. Currently, the most widely used metabolomics techniques are nuclear magnetic resonance (NMR), gas chromatography-mass spectrometry (GC–MS), and liquid chromatography-mass spectroscopy (LC–MS)^14^. Among them, LC–MS has become the main technical platform for metabolomics analysis due to its advantages of high throughput, high sensitivity and specificity, wide range of application, short detection cycle, simultaneous detection of multiple indicators, and high accuracy^15^.

In our laboratory, we have previously analyzed the metabolomic profiles in skeletal muscle and urine for the effects of aerobic exercise intervention on the CUMS-induced depression using a rodent model^16,17^. The pathogenesis of depression is complex and involves alterations in the functioning of multiple systems. As a commonly used sample for clinical diagnostics and metabolomic analysis, the variety of metabolites in serum samples will be relatively comprehensive and stable, reflecting the metabolic changes in the body caused by external stimuli at an overall level. LC–MS/MS metabolomics techniques enable the detection of a more comprehensive and representative range of metabolites, contributing to the multi-target characterisation of the mechanisms of exercise intervention in disease. The aim of this study was to reveal the modulation of the serum metabolic profile of depressed rats by aerobic exercise using an LC–MS/MS metabolomics approach, and to provide a new research strategy for the in-depth study of the antidepressant effects and potential mechanisms of exercise.

## Results

### Behavior tests

After 21 days of exposure to CUMS, the body weight, sucrose preference rate, and the number of crossings and standings of CUMS-treated rats were significantly decreased compared with the normal control group (C), whilst no significant difference was found between groups before CUMS modeling, indicating the rats under CUMS had developed depression-like symptoms.

**Figure 1:**
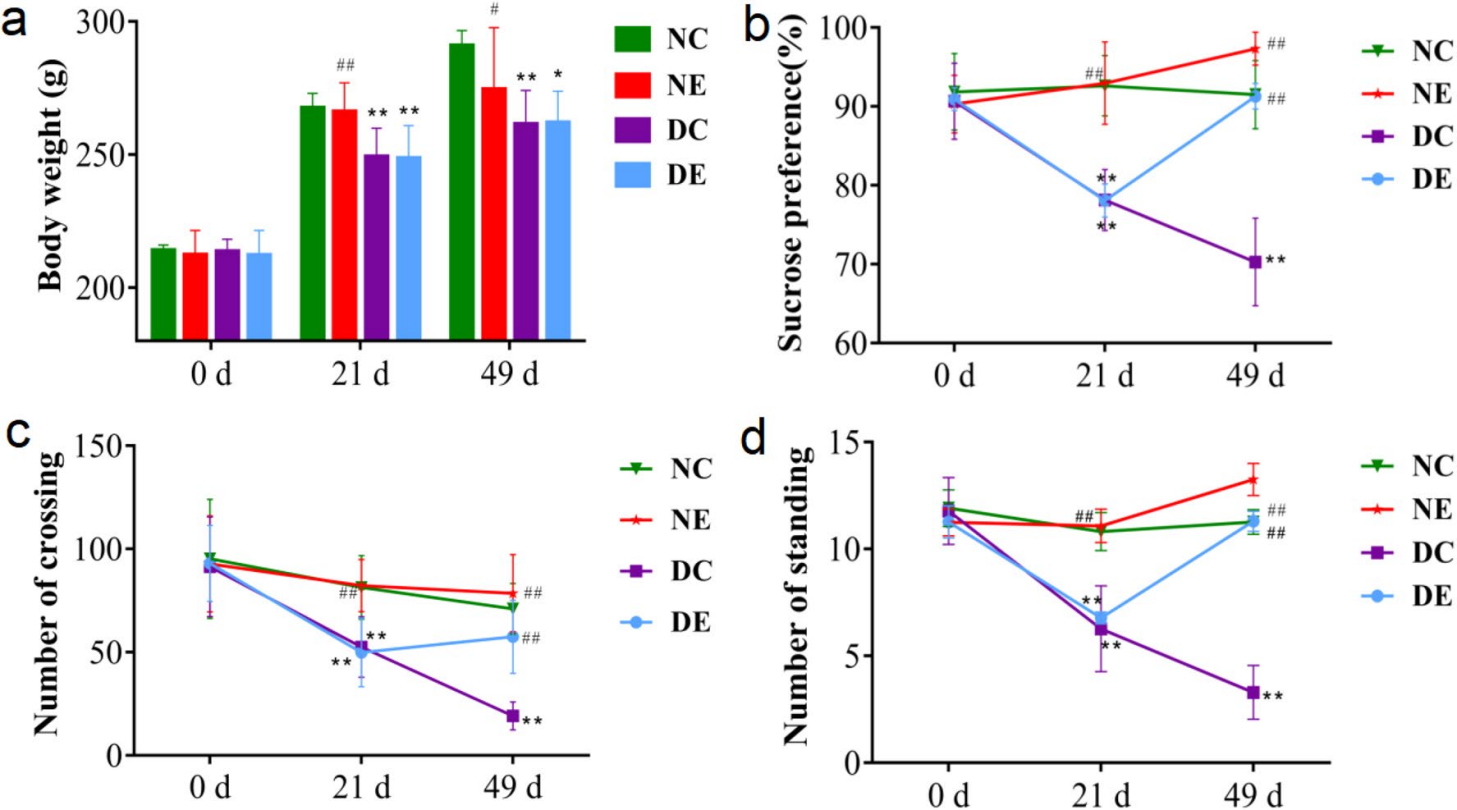
Effects of CUMS and aerobic exercise on behavioral indicators. (**a**) body weight; (**b**) sucrose preference rate; (**c**) number of crossing in open field test; and (**d**) number of standing in open field test of rats during the 49-day experiment. Data are presented as mean ± SD (n = 8 in each group). *p < 0.05, **p < 0.01 versus NC; #*p* < 0.05, ##*p* < 0.01 versus DC. *CUMS* chronic unpredictable mild stress.

At the end of the 28-day aerobic exercise intervention, sucrose preference rate, and the number of crossings and standings were significantly higher in group DE compared to group DC, indicating beneficial effects of the aerobic exercise on the depression-like behavior (Fig. 1). It is worth noting that after 28 days of aerobic exercise, the body weight of rats in the DE group was still significantly lower compared to the NC group, suggesting that aerobic exercise did not alleviate the weight loss caused by CUMS modelling, which might relate to increased energy consumption during exercise. In addition, group NE showed a trend of lower body weight and higher sucrose preference rate and the number of crossings and standings compared to group NC, although the differences were not statistically significant. This suggests that the exercise in normal rats did not cause additional stress.

### Multivariate statistical analysis of LC–MS/MS data

Data processing in metabolomics usually involves grouping, classifying, and discriminating the collected data, with the main means including unsupervised and supervised methods, and then studying the metabolite change patterns and mechanisms after the organism is perturbed^18^.

**Figure 2:**
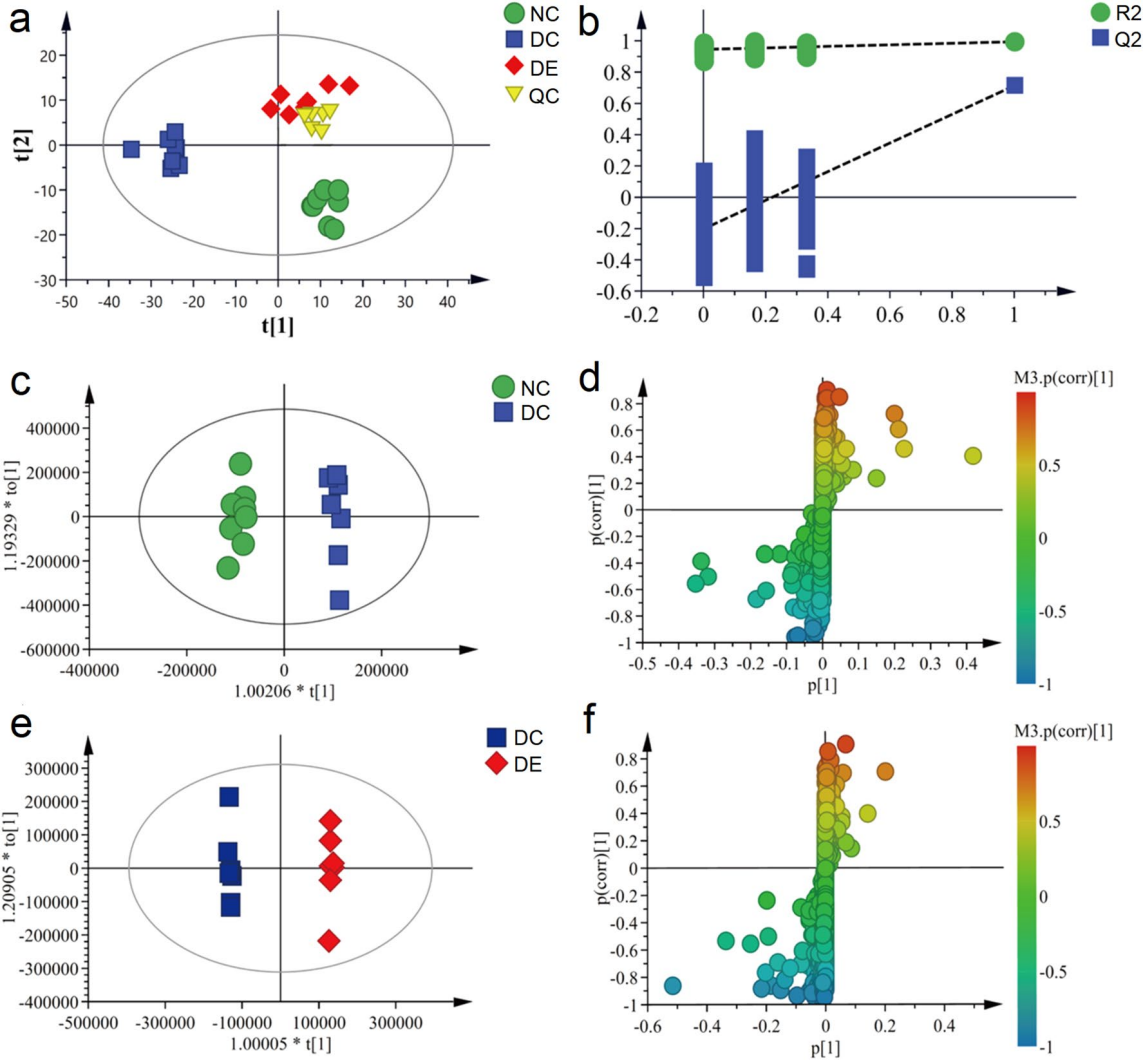
Multivariate data analysis from LC–MS/MS. (**a**) PLS-DA score plots from group NC, group DC, group DE, and QC; (**b**) PLS-DA model validation diagram; (**c**) OPLS-DA score plots from group NC and group DC; (**d**) S-plot of group NC and group DC; (**e**) OPLS-DA score plots from group DC and group DE; (**f**) S-plot of group DC and group DE. *QC* Quality control, *PLS-DA* partial least squares discriminant analysis, *OPLS-DA* Orthogonal partial least squares discriminant analysis.

To distinguish the serum metabolic profiles of each group visually, the present study firstly used an unsupervised PCA model to perform a preliminary analysis of all sample data to eliminate outliers. Then, a supervised PLS-DA model analysis was performed on each group of sample data to obtain PLS-DA score scatter plots and model validation plots (Fig. 2a,b). As shown by the PLS-DA score plot, the metabolic profiles of the three groups showed a certain clustering trend, with the DE group was clearly separated from the DC group, indicating that aerobic exercise intervention for depression resulted in significant changes in the metabolic profiles of the depressed rats. In addition, the QC samples of PLS-DA were clustered together, indicating that the system and method were stable.

To verify the reliability of the model, permutation test (n = 200 times) was performed on the above model data, and the results showed that the R2 and Q2 parameters were close, the model slope was large, and the intercept with the vertical axis was negative, which indicate that the model was robust and did not have overfitting phenomenon.

To find differential metabolites associated with depression and exercise to improve depressive symptoms, OPLS-DA analysis was first performed on the data from the NC and DC groups to remove experimentally irrelevant information and to screen for differential variables associated with depressive illness. The OPLS-DA score plot showed significant separation between the DC and NC groups (Fig. 2c), indicating that depression affected the serum metabolic profile. The variables with the greatest contribution to variance were then screened by combining VIP > 1 in the S-Plot plot (Fig. 2d), and significant difference (*p* < 0.05) was found by the independent samples t-test. Next, OPLS-DA analyses were performed on the DE and DC groups to screen for differential variables associated with the improvement in depression by the exercise intervention. The OPLS-DA score plot showed that the group DC and group DE were also significantly separated (Fig. 2e), indicating that the aerobic exercise had a modulatory effect on the serum metabolic profile in the depression model rats and combined with S-Plot plots (Fig. 2f) to screen for the most contributing differential variable.

### Analysis of differential metabolites in serum

**Figure 3:**
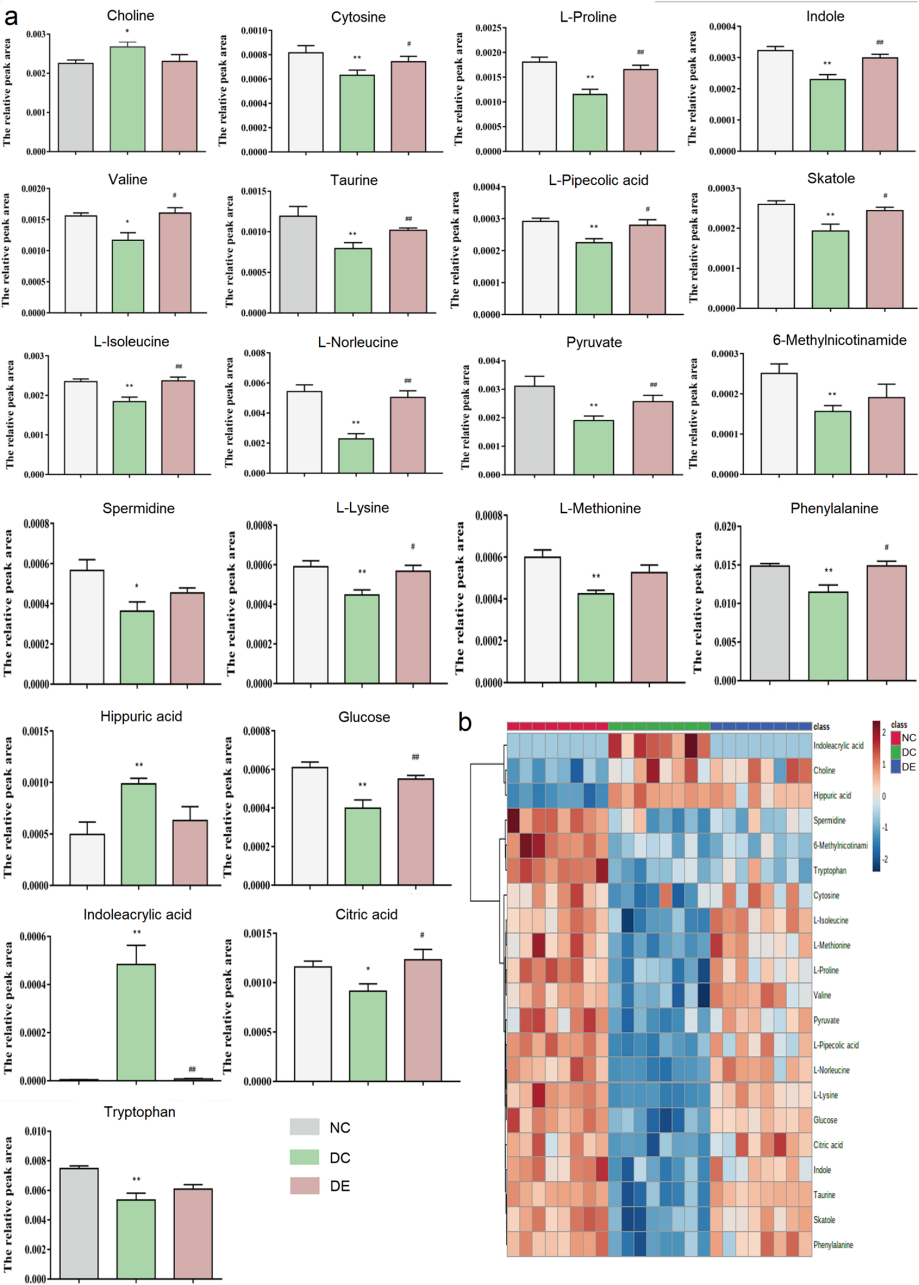
(**a**) Comparison of the relative peak areas of differential metabolites. All data were expressed as mean ± SD (n = 8). *p < 0.05, **p < 0.01 versus NC; #*p* < 0.05, ##*p* < 0.01 versus DC. (**b**) The relative content of the heat map of differential metabolites in serum samples of rats under CUMS. *CUMS* chronic unpredictable mild stress.

A total of 21 differential metabolites related to depression were identified by OPLS-DA analysis of serum sample data, of which 15 differential metabolites were modulated after exercise (Table 1). The relative peak area levels of these metabolites are shown in the histogram (Fig. 3a). To visualize the overall trend of all differential metabolites in each sample, a hot spot plot of their relative levels was presented (Fig. 3b).

**Table 1:** Differential metabolites in the serum of CUMS rats with or without aerobic exercise.

No	Metabolites	T_R_ (min)	m/z	VIP	DC/NC	DE/DC
1	Choline	1.259	103.09993	2.26815	↑*	–
2	Cytosine	1.591	111.04337	1.04266	↓**	↑^#^
3	L-Proline	1.332	115.06341	2.70859	↓**	↑^##^
4	Indole	5.819	117.05783	1.20876	↓**	↑^##^
5	Valine	1.585	117.07905	1.95096	↓*	↑^#^
6	Taurine	1.305	125.0145	1.07995	↓**	↑^**##**^
7	L-Pipecolic acid	1.047	129.07892	1.05517	↓**	↑^#^
8	Skatole	5.819	131.07336	1.04436	↓**	↑^#^
9	L-Isoleucine	1.599	131.09459	1.41987	↓**	↑^##^
10	L-Norleucine	3.091	131.0946	5.23361	↓**	↑^##^
11	Pyruvate	1.601	87.0359	1.37331	↓**	↑^**##**^
12	6-Methylnicotinamide	1.279	136.06354	1.04442	↓**	**–**
13	Spermidine	0.988	145.15775	1.36626	↓*	**–**
14	L-Lysine	1.048	146.10539	1.34038	↓**	↑^#^
15	L-Methionine	1.595	149.05102	1.21061	↓**	–
16	Phenylalanine	1.791	165.07884	2.33672	↓**	↑^**#**^
17	Hippuric acid	6.285	179.05806	1.47445	↑**	–
18	Glucose	1.249	180.06327	1.74516	↓**	↑^##^
19	Indoleacrylic acid	5.819	187.06314	7.86256	↑**	**↓**^##^
20	Citric acid	1.596	192.02688	1.90164	↓*	↑^#^
21	Tryptophan	5.819	204.08966	5.95274	↓**	–

*p < 0.05, **P < 0.01 versus NC; #*p* < 0.05, ##*p* < 0.01 versus DC; ↑: Increase, ↓: Decrease.

After 49 days of CUMS modeling, the levels of 21 different metabolites were altered differently in group NC and group DC. Of these, the serum levels of 18 different metabolites, including cytosine, L-proline, indole, valine, taurine, L-pipecolic acid, skatole, L-isoleucine, L-norleucine, pyruvate, 6-methylnicotinamide, spermidine, L-lysine, L-methionine, phenylalanine, glucose, citric acid, and tryptophan, were significantly lower decreased, and the levels of 3 different metabolites, choline, hippuric acid, and indoleacrylic acid, were significantly increased. The aerobic exercise modulated 15 of these metabolites, including cytosine, L-proline, indole, valine, taurine, L-pipecolic acid, skatole, L-isoleucine, L-norleucine, L-lysine, phenylalanine, glucose, indoleacrylic acid, citric acid, and pyruvate, as indicated by that in the DE compared with DC (Table 1).

### Pathway analysis of serum differential metabolites

**Figure 4:**
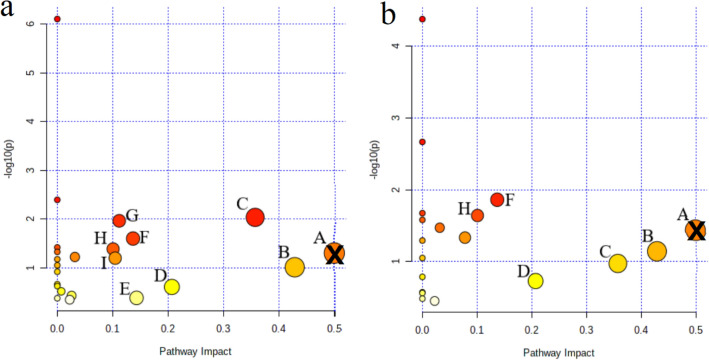
Summary diagram of pathway analysis with Met-PA. (**a**) The metabolic pathways associated with depression; (**b**) Regulation of metabolic pathway of aerobic exercise. A: phenylalanine, tyrosine and tryptophan biosynthesis ( ×); B: taurine and hypotaurine metabolism; C: phenylalanine metabolism; D: pyruvate metabolism; E: tryptophan metabolism; F: citrate cycle (TCA cycle); G: arginine and proline metabolism; H: glycolysis/gluconeogenesis; I: cysteine and methionine metabolism. Each circle represents one metabolic pathway; the size of the circle and shades of color are positively correlated with the impact on the metabolic pathway.

A total of 21 differential metabolites were found to be associated with depression using the LC–MS/MS method and 15 differential metabolites were affected by the aerobic exercise. The 15 differential metabolites were imported into the MetaboAnalyst 5.0 database for metabolic pathway analysis to obtain the pathway impact distribution map and pathway enrichment map (Fig. 4). A total of nine metabolic pathways significantly associated with depression were screened according to impact value > 0.1 (Fig. 4a), mainly including phenylalanine, tyrosine and tryptophan biosynthesis, taurine and hypotaurine metabolism, phenylalanine metabolism, pyruvate metabolism, tryptophan metabolism, citrate cycle (TCA cycle), arginine and proline metabolism, glycolysis/gluconeogenesis, cysteine and methionine metabolism. The aerobic exercise affected six of these metabolic pathways (Fig. 4b), mainly including phenylalanine, tyrosine and tryptophan biosynthesis, taurine and hypotaurine metabolism, phenylalanine metabolism, pyruvate metabolism, citrate cycle (TCA cycle), glycolysis/gluconeogenesis. Since animals do not have complete pathways for the synthesis of tyrosine, phenylalanine and tryptophan, which are synthesized only by bacteria, other microorganisms and plants^19^, we excluded the metabolic pathways of phenylalanine, tyrosine and tryptophan biosynthesis from further analysis and discussion in this report. Therefore, we have identified 8 metabolic pathways associated with depression, and aerobic exercise may exert antidepressant effects by modulating 5 of them.

### Correlation analysis of behavioral indicators of depression and levels of serum differential metabolites

**Figure 5:**
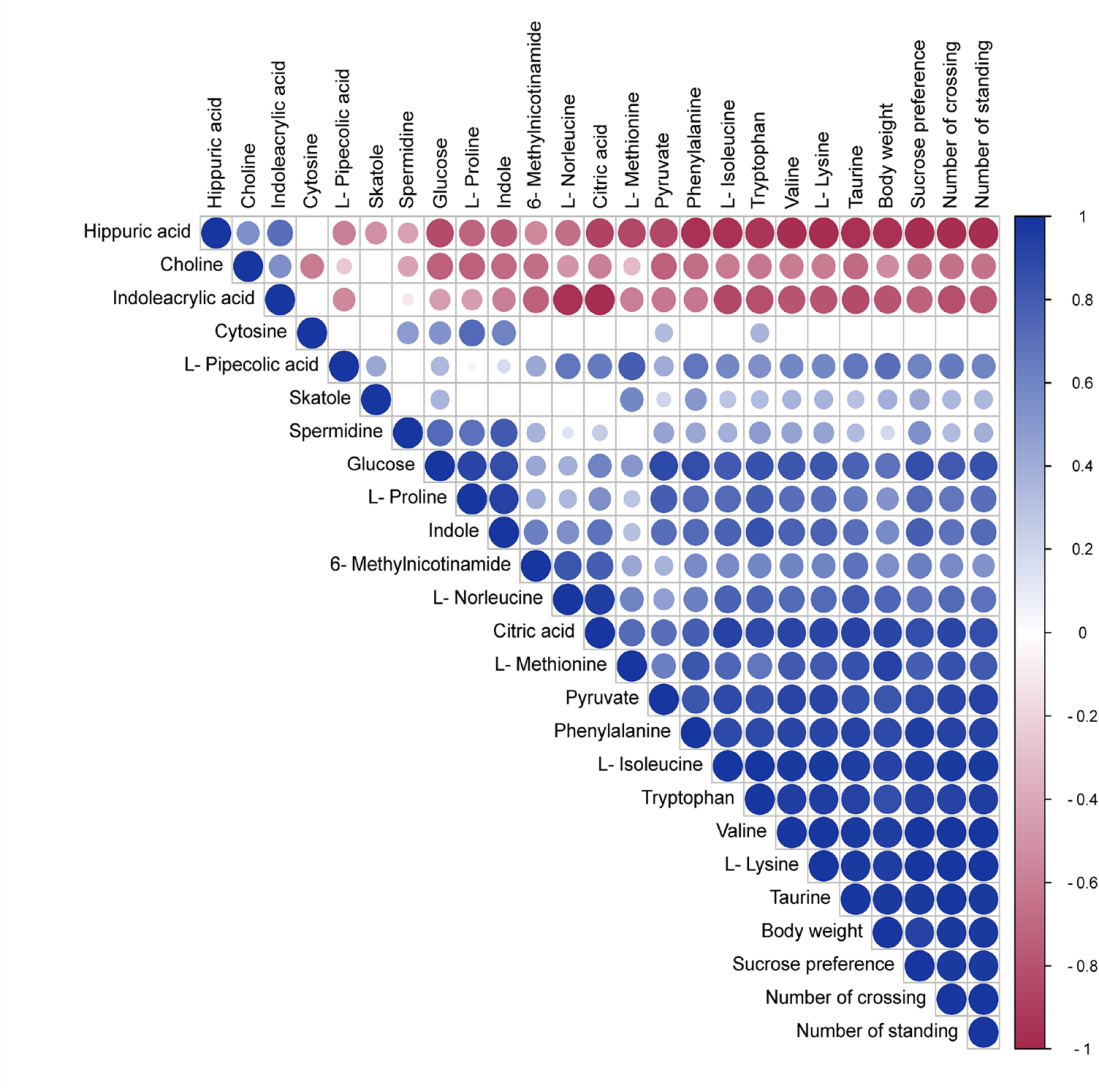
Heat map of the correlation between differential metabolites and behavioral indicators based on Spearman's correlation analysis.

In the heat map of the correlation, the size and color of the circles represent the degree of correlation between the indicators, and blue is positive and red is negative. Correlation coefficients range from 1.0 (maximum positive correlation) to − 1.0 (maximum negative correlation), with 0 indicating no correlation. According to the threshold |r|> 0.5 combined with *p* < 0.05 indicates a significant correlation between the two indicators. As shown in Fig. 5, the spearman's correlation analysis shows that the body weight, sucrose preference rate, and the number of crossings and standings were significantly and negatively correlated with levels of Hippuric acid and Indoleacrylic acid, and positively correlated with levels of Glucose, Proline, Citric acid, L-Methionine, Pyruvate, Phenylalanine, L-Isoleucine, Tryptophan, Valine, L-Lysine, and Taurine.

## Discussion

In this study, a CUMS rat model was successfully established through 21 days of chronic unpredictable mild stress combined with individual housing. After 28 days of aerobic exercise intervention, the number of crossings and standings in the OFT and sucrose intake in the SPT were significantly increased in CUMS rats, suggesting that aerobic exercise resulted in a significant improvement in CUMS-induced depression. Furthermore, we analyzed the serum metabolic profile of the antidepressant effect of the aerobic exercise based on the metabolomic approach of LC–MS/MS in CUMS model rats, to clarify the specific metabolic pathways associated with the antidepressant effect of aerobic exercise. A total of 21 serum biomarkers associated with depression were identified, and the aerobic exercise intervention significantly regulated 15 of them. Enrichment analysis of metabolic pathways revealed that the antidepressant effect of the aerobic exercise was mainly related to the markers of 5 metabolic pathways in amino acid metabolism and energy metabolism. Spearman's correlation analysis showed that the body weight, sucrose preference rate, and the number of crossings and standings were strongly correlated with the levels of differential metabolites in serum. This study provides a new research strategy to further explore the role and mechanisms of exercise in improving depression.

Amino acids are the basic units of protein and the precursors of neurotransmitters in the central nervous system, and their metabolites are involved in the body's immune response^20^. The synthesis of neurotransmitters in the brain is influenced by amino acid levels^21,22^. Amino acid metabolism is an important metabolic pathway in the body, and different amino acids play different functions in the pathogenesis of depression. Studies suggest that abnormal amino acid metabolism may be a common manifestation of depression and that disturbed amino acid levels may serve as a clinical trait-biomarker for depression^23,24^. Phenylalanine is capable of producing tyrosine via phenylalanine hydroxylase, and tyrosine can be further metabolized to catecholamine neurotransmitter precursors via tyrosine hydroxylase, that is closely associated with depression^25^. The significantly lower level of phenylalanine in the DC group may affect the synthesis of tyrosine and catecholamine neurotransmitters, resulting in a decrease of dopamine, which is involved in the pathogenesis of depression. In this study, aerobic exercise significantly reversed the decrease in serum phenylalanine levels induced by CUMS in rats. Fatigue is a common symptom of depression. Valine and isoleucine are both branched-chain fatty acids (BCAAs) that cross the blood–brain barrier and compete with tryptophan, the precursor of 5-HT. When levels of BCAAs are reduced, they interfere with the release of 5-HT in the brain, resulting in central fatigue^26^. In the present study, serum levels of valine and isoleucine were significantly lower in the DC group of rats. And after aerobic exercise, the levels of valine and isoleucine were increased significantly, suggesting that aerobic exercise may improve the symptoms of fatigue in depression by modulating the levels of valine and isoleucine. Taurine is one of the most abundant free amino acids in the central nervous system^27^. It has various important physiological functions as a neuromodulator and an antioxidant, such as its ability to reduce inflammation and oxidative stress-mediated damage as shown in several disease models^28^. Taurine has been shown to have a protective effect on brain development, effectively improving learning ability and memory, and may play an important role in neurodegenerative diseases such as depression and Alzheimer's disease^29,30^. Perry et al. reported that families that suffer from a hereditary taurine deficiency have a tendency to develop depression^31^. Murakami et al. offered the first case to demonstrate that chronic ingestion of taurine-supplemented diet had an antidepressant-like effect^32^. Wu et al^33^. found that depression-like behaviors were significantly improved by pre-administration of taurine to CUMS rats, and its antidepressant mechanism may be involved in regulating the hypothalamo-pituitary adrenal (HPA) axis function, promoting neurogenesis, cell proliferation, neuronal survival, and growth in the hippocampus. In the present study, we found that taurine levels in the serum of depressed rats showed a significant decrease, while their levels increased significantly after aerobic exercise. In conclusion, these changes in metabolites suggest that amino acid metabolism plays an important role in the antidepressant effects of aerobic exercise.

Abnormalities in metabolic pathways such as pyruvate metabolism, TCA cycle, and glycolysis/gluconeogenesis are mainly related to the disorder of energy metabolism. Many animal experiments have demonstrated that the pathophysiology of depression is closely related to a dysfunction of energy metabolism^34,35^. Glucose is the main energy-providing molecule in the body. Through the glycolytic pathway, glucose can be converted to pyruvate^36^. Pyruvate is the end product of glycolysis, and can be converted to acetyl coenzyme A via the pyruvate dehydrogenase complex, which is used in the synthesis of citrate and is further involved in the tricarboxylic acid (TCA) cycle to generate ATP^37^. A decreased level of pyruvate suggests a disturbance of glycolysis/gluconeogenesis pathways, and may further lead to TCA cycle dysfunction and depressive symptoms. In our present study, compared to the NC group, the levels of pyruvate, citrate, and glucose in the serum of the DC group were significantly lower. It appears that the aerobic exercise could have affected the energy metabolism by increasing the levels of pyruvate, citrate, and glucose in the serum.

Our team has previously identified and confirmed metabolic markers in urine samples that were associated with aerobic exercise for improving depression in biological samples from CUMS rats using the ^1^H NMR technique, such as glutamine, acetone, pyruvate, creatine, and trigonelline^17^; and in the soleus muscle samples, propylene glycol, lactate, anserine, choline phosphate, and alpha-glucose in the gastrocnemius; alanine, creatinine lactate, anserine, choline, choline phosphate, histidine, and inosine^16^. This study used LC–MS/MS technique and found that the aerobic exercise modulated the levels of 15 depression-related serum metabolites including cytosine, L-proline, indole, valine, taurine, L-pipecolic acid, skatole, L-isoleucine, L-norleucine, L-lysine, phenylalanine, glucose, indoleacrylic acid, citric acid, and pyruvate. Interestingly, we observed that despite the different specimens we examined, the majority of the depression-related metabolites modulated by the aerobic exercise were mainly related to energy and amino acid metabolism. Moreover, the metabolic pathway analysis based on the identified metabolites revealed that the differential metabolites in the urine sample were mainly involved in energy metabolism, amino acid metabolism, and gut microbial metabolism. The differential metabolites in gastrocnemius, soleus muscle, and serum samples were mainly related to energy metabolism and amino acid metabolism. Thus, future research could focus on how exercise specifically modulates energy metabolism and amino acid metabolism in manifestation of the antidepression effects for a more comprehensive validation.

In this study, aerobic exercise interventions had shown positive effects of improving the depressive symptoms in rats under CUMS. In addition, we used the LC–MS/MS technique to investigate the metabolic regulatory pathways in serum that were related to the antidepressant effect of aerobic exercise. The results showed that a total of 21 serum metabolites associated with depression were identified, 15 of which were significantly modulated by the aerobic exercise. The correlation analysis also indicated that changes in serum levels of metabolites in CUMS rats were closely related to behavioral disorders. Combined with the results of ^1^H NMR experiments in our previous reports, the effects of aerobic exercise on depression were mainly related to the changes in pathways of energy metabolism and amino acid metabolism. The current study provides not only potential diagnostic biomarkers for depression and antidepressant effects of aerobic exercise, but also a metabolomic approach to investigate the underlying mechanisms of exercise intervention in the management of depression.

## Methods

### Animals and ethic statement

32 Sprague–Dawley male rats (8 weeks old, body mass 210 ± 15 g (mean ± SD) were obtained from the Experimental Animal Center of Beijing Weitong Lihua Technology Co. Ltd. (Animal License Number: SCXK, Beijing, China, 2016-0006). The minimum number of animals required to achieve adequate statistical power using a two-way ANOVA with repeated measures (for behavioral tests) was estimated using G*Power 3.1.9.7. With the assumptions of Effect Size 0.25, alpha 0.05, power 0.9, 4 groups and 5 measurements, the minimum number required was 32, with 8 in each group. The rats were housed in cage, with four in each cage, for 7 days adaption to the environment before commencement of the experiment. The animal house was under controlled conditions of 12 h light–12 h dark cycles, temperature of 22–27 °C, and relative humidity of 30–70%, and the animals had free access to rodent food and water.

The research obtained approval by the Committee of Scientific Research at Shanxi University (CSRSX). All animal experiments were conducted following the NIH Guidelines for Care and Use of Laboratory Animals (U.S.A) and the Prevention of Cruelty to Animals Act (1986) of China, and all experimental procedures tried to minimize the suffering of experimental animals. This study was carried out in compliance with the ARRIVE guidelines.

### Establishment of the depression model

**Figure 6:**
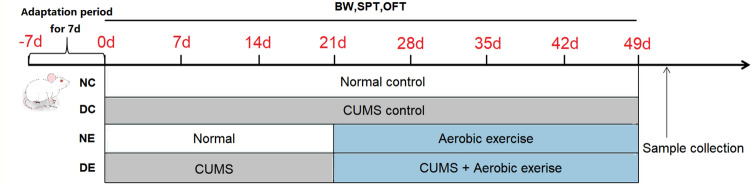
Experimental program and behavior test schedule. *BW* body weight, *SPT* sucrose preference test, *OFT* open field test.

The animals were randomly divided into four groups (n = 8 in each): normal control (NC), normal with aerobic exercise (NE), CUMS control (DC), and CUMS with aerobic exercise (DE). The modeling process followed the method of Willner et al.^38^. In this study, to induce depression under CUMS, the modeling period lasted for 21 days, and each rat was randomized to receive one stimulus per day. The applied stressors are outlined (Table 2). Group DE received exercise from the day 22 for a total of 28 days, and continued to receive CUMS stimulation during the exercise intervention period. Some studies have shown that rats housed in single cage can cause learning and memory impairment and depression-like behaviors^39^, so we also use individual housing as a stimulus. Untreated rats (NC, NE) were still housed together, whereas those intended for CUMS (DC, DE) were individually housed in a smaller cage. During the experiment, behavioral tests, including body weight measurement (BW), sucrose preference test (SPT), and open field test (OFT) were conducted every 7 days to evaluate the depressive symptoms of each group of rats. The behavioral tests followed the methods previously described in our laboratory^17,40^. The experimental flow chart is shown in the figure (Fig. 6).

**Table 2:** The applied stressors of CUMS. CUMS; chronic unpredictable mild stress.

Stressor	Description
Water deprivation	24 h of water deprivation
Food deprivation	24 h of food deprivation
Ice bath	Rats placed on glass jar with ice water at 4 °C to swim for 5 min
Ultrasonic stimulation	Ultrasound stimulation (60 W) lasted for 3 h
Thermal stimulus	Rats placed on electric thermostat at 45 °C for 10 min
Electric shocks	Rats were placed in a plantar electric box with a voltage of 32 V, and the electric shock was given every 10 s for 2 s for a total of 10 times
Tail clamp	The tail of the rats was clamped with a long tail clip for 2 min (the rats whined)
Restraint	Rats was restrained in transparent plastic bottles for 3 h and returned to their original cage afterward
Day and night reversal	Rats placed on the dark space during the day and in the bright space at night for 12 h each

### Aerobic exercise

**Figure 7:**
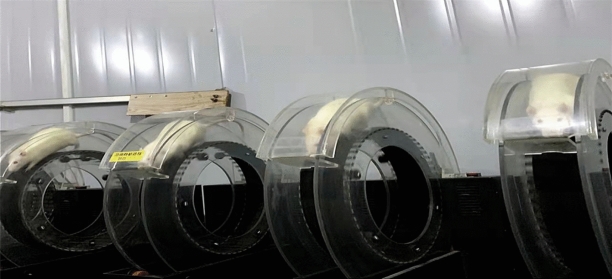
The wheeled motorized treadmill for aerobic exercise.

The aerobic exercise in this experiment utilized wheeled motorized treadmills for running (Fig. 7). To reduce the stress associated with unfamiliar exercise, rats were familiarized with the treadmill run for three consecutive days, with 15 min per day, before starting the formal exercise intervention. The running speed was 3 m/min for the first 5 min, 5 m/min for the next 5 min, and 8 m/min for the last 5 min. Rats in the NE and DE groups exercised on the YLS-15A Wheeled motorized treadmill, starting from the day 22 of CUMS (Beijing Zhongshidichuang Science and Technology Development Co. Ltd.). The aerobic exercise protocol had been described previously^16,17^. Briefly, rats in the DE and CE groups exercised on the treadmill for 30 min per day, 5 times per week for four weeks. The treadmill speed started from 3 m/min for 5 min, increased to 5 m/min for 5 min, then to 8 m/min for 20 min. No additional stimuli were applied to the rats during this procedure. During exercise, rats in the NC and DC groups were also placed in the same room to control for environmental factors.

### Sample collection and preparation

After the last aerobic exercise, BW, SPT, and OFT were performed on all rats. Then all rats were deeply anesthetized after 12-h of fasting. Blood sample was collected through the abdominal aorta, left for 30 min, centrifuged at 4 °C and 3500 rpm for 10 min, and the serum was separated and divided into EP tubes of about 2 mL each, and then stored in a deep freezer at − 80 °C before the LC–MS/MS analysis.

### Metabolomics analysis by LC–MS/MS

Sample preparation: The serum specimens were taken from the − 80 °C freezer and thawed at 4 °C. 100 μL of serum was added to 200 µL acetonitrile containing 0.1% formic acid, and the mixture was vortexed for 2 min. After being centrifuged at 13,000 rpm for 20 min at 4 °C, each supernatant was filtered through a 0.22 mm membrane filter and collected in a UPLC vial for LC–MS analysis.

Metabolic profiling data acquisition: Metabolic profiling was first based on non-targeted analysis by a Thermo-Fisher ultra-performance liquid chromatography system (UHPLC) (Thermo-Fisher, USA) coupled to a Q Exactive Orbitrap-MS (Thermo-Fisher, USA). Chromatographic separation was carried out on a Waters ACQUITYUPLC HSS T3 column (2.1 × 100 mm, 1.8 µm) maintained at 40 °C. The mobile phase consisted of A (water with 0.1% formic acid) and B (acetonitrile with 0.1% formic acid) solutions. The eluting gradient applied were: 0–2 min, 2% B; 2–3 min, 2–35% B; 3–17 min, 35–70% B, 17–18 min, 70% B; 18–29 min, 70–98% B; 29–31 min, 98% B; 31–33 min, 98–2% B; 33–35 min, 2% B. The flow rate was 0.2 mL/min and the injection volume was 5 μL. The mass spectrometric data were collected using the mass spectrometer in both positive and negative ion modes with the following parameters: capillary temperature, 320 °C; heater temperature, 300 °C; sheath gas velocity, 35 arb; auxiliary gas flow rate, 10 arb; scan range, m/z 100–1500. Quality control (QC) samples were obtained by combining equal aliquots of serum, which were processed in the same way as samples. Throughout the sample analysis, 1 shot of the QC sample was injected after every 4 shots of the sample to monitor the stability of the LC–MS/MS system.

### Data processing

The raw data from the LC–MS/MS were exported using the Xcalibur workstation (Thermo Fisher Scientific Inc., Waltham, Ma, USA) and imported to Compound Discoverer 3.1 (Thermo Fisher, USA) to obtain the matched and aligned peak data. The setting parameters were as follows: mass range 100–1500 Da; mass tolerance 5 ppm; RT tolerance 0.05 min; and S/N threshold 1.5. Then, the data were transferred into Excel for peak area normalization after being processed by Compound Discoverer 3.1.

Thereafter, the normalized peak area data were transferred into SIMCA-P software (ver. 14.1, Umetrics, Umea, Sweden) for the multivariate statistical analysis. First, the principal component analysis (PCA) of the normalized data was used to identify the degree of dispersion between NC group and DC group, and the outliers were eliminated. Next, partial least squares discriminant analysis (PLS-DA) was used to distinguish the differences in metabolic profiles between the NC, DC and DE groups. Orthogonal partial least squares discriminant analysis (OPLS-DA) was used to find differential metabolites among NC group and DC group as well as DC group and DE group separately. Finally, ANOVA analysis was performed on the metabolites using IBM SPSS (ver. 23.0) software, with VIP > 1 and *p* < 0.05 as differential metabolites. The database sources of HMDB (http://www.hmdb.ca), Pubchem (https://pubchem.ncbi.nlm.nih.gov/), KEGG (http://www.kegg.jp) and the related literature were queried with the exact masses of the metabolites to identify the differential metabolites. Finally, the identified differential metabolite data were enriched by MetaboAnalyst 5.0 (https://www.metaboanalyst.ca/) for pathway analysis.

### Statistical analysis

Statistical analyses were performed by two-way ANOVA followed by Dunnett’s multiple comparisons using GraphPad Prism 7.0 (GraphPad Software, Inc., La Jolla, CA, USA) and SPSS statistics 23.0 software (Chicago, IL, USA). *p* < 0.05 was considered statistically significant.
